# Horse Preferences for Insolation, Shade or Mist Curtain in the Paddock under Heat Conditions: Cardiac and Behavioural Response Analysis

**DOI:** 10.3390/ani11040933

**Published:** 2021-03-25

**Authors:** Iwona Janczarek, Anna Stachurska, Izabela Wilk, Anna Wiśniewska, Monika Różańska-Boczula, Beata Kaczmarek, Jarosław Łuszczyński, Witold Kędzierski

**Affiliations:** 1 Department of Horse Breeding and Use, Faculty of Animal Sciences and Bioeconomy, University of Life Sciences in Lublin, 20-950 Lublin, Poland; iwona.janczarek@up.lublin.pl (I.J.); izabela.wilk@up.lublin.pl (I.W.); anna.wisniewska@up.lublin.pl (A.W.); 2 Department of Applied Mathematics and Computer Science, Faculty of Production Engineering, University of Life Sciences in Lublin, 20-950 Lublin, Poland; monika.boczula@up.lublin.pl; 3 Sub-Department of Internal Diseases of Farm Animals and Horses, Faculty of Veterinary Medicine, University of Life Sciences in Lublin, 20-612 Lublin, Poland; beatakaczmar1@gmail.com; 4 Department of Genetics, Animal Breeding and Ethology, Faculty of Animal Science, University of Agriculture in Cracow, 30-059 Cracow, Poland; jaroslaw.luszczynski@urk.edu.pl; 5 Department of Biochemistry, Faculty of Veterinary Medicine, University of Life Sciences in Lublin, 20-950 Lublin, Poland; witold.kedzierski@up.lublin.pl

**Keywords:** horse, welfare, cardiac variables, emotional arousal, behaviour, shade, sun, mist curtain

## Abstract

**Simple Summary:**

Most housing guidelines for horses recommend providing access to shade in paddocks and pastures on hot days. Some paddocks are equipped with mist curtains to enable horse cooling. However, earlier studies on horse behaviours have shown that the provision of shade is not an absolute care requirement. The use of mist curtains by horses has not been scientifically studied until now. The objective of the current study was to determine which area in a paddock (insolated, shaded, or water sprayed) is most often chosen by horses during a short, 45 min stay in a hot environment (29–32 °C, 42.0% humidity) and whether horses’ behaviour and emotional arousal (expressed by cardiac variables) are related to exposure to factors prevailing in these places. The study found that the horses’ preferences regarding the conditions in the paddock were not evident, although particular horses differed in this respect. The horses did not show symptoms of overheating while staying in the sun. They were more relaxed in insolated and water sprayed areas than in the shade. The free choice of different areas provided in paddocks seems to enable horses to maintain body temperature and emotional arousal at similar levels.

**Abstract:**

The horse’s welfare and, consequently, the emotional arousal may be connected with stressful environmental conditions. This study aimed to determine whether horses show behavioural or physiological symptoms of thermal discomfort and if their behaviour and cardiac parameters are related to freely chosen insolated (IS), shaded (SH), or water sprayed (with a mist curtain (MC)) areas in a paddock under heat conditions (29–32 °C, 42.0 ± 1.5% humidity). Twelve adult horses freely moving in the paddock were studied during a 45 min solitary turnout. Six cardiac variables, locomotor, and non-locomotor activities as well as rectal temperature before and after the test were monitored with regard to the area of staying. Horses did not show clear preferences regarding the time spent in IS, SH, and MC, although preferences of particular horses differed considerably. When staying under IS and MC conditions, the horses showed a higher level of relaxation compared to SH. Horses did not exhibit symptoms of thermal discomfort while staying in the sun. Free choice between the three areas differing in environmental conditions could be a crucial factor in maintaining body temperature as well as emotional arousal at similar levels. Thus, the provision of a shade and mist curtain in paddocks seems to be reasonable.

## 1. Introduction

Horses possess a high metabolic capacity and, simultaneously, a relatively small surface area for dissipation of heat. Thus, they suffer a disadvantage compared to many other species. Knowledge of horse needs is crucial in providing welfare to these animals. After a long period of domestication and adaptation to staying mainly in the stable, it is unclear whether horses possess a capacity to enhance thermoregulation and thus whether they choose insolated or chilling areas under heat conditions when freely moving in the paddock. The thermoregulation of horses is mainly dependent on evaporative heat loss from sweating [1]. Horses sweat to maintain body core temperature [2]. The sweating starts when vasodilatation is not sufficient, and the skin blood flow cannot increase enough to transfer the heat from the body core to the skin surface [3]. Under thermally stressful conditions, particularly during strenuous exercise, sweat losses may amount to ten litres per hour, which constitutes two-thirds of the metabolic heat load. The remaining heat increases the core temperature and is dissipated mainly by the respiratory system [4]. Horses demonstrate a high degree of tolerance to environmental heat load; however, when exercised under severely hot and humid conditions, hyperthermia may develop in them [5]. During exercise, the conversion of energy is relatively inefficient since only 20% of the metabolism in the muscle cells is used for work and up to 80% is transformed into heat [6]. 

The thermoregulation of the horse is considered particularly in terms of athletic exercise when effort is high and heat stress may develop [7]. To improve the welfare of competing horses and prevent overheating, scientific studies have been conducted, e.g., on the effectiveness of such methods as the application of cold water [8] or clipping the winter coat [6]. Takahashi et al. [9] found that showering with tap water was the most effective method to decrease the temperature in horses. The horse body temperature is also considered in the context of assuring good welfare conditions affecting the performance in different horses at rest, during effort, and in transport [10,11]. Horses released freely into a paddock have mainly been studied with regard to their relaxation [12], behaviour [13,14], and preferences for the time spent out of the stable [15]. Open stables and, particularly, active stables equipped with functional elements located in different places inside and outdoor induce locomotor activity and enable demonstrating preferences by horses [16]. 

It is commonly believed that shady places are indispensable for horses during hot weather, hence providing access to shade is recommended by most guidelines. Furthermore, mist curtains are sometimes installed in the paddock to enable immediate cooling. Moreover, horse owners often claim that horses rarely hide in the shade on hot days. According to Cymbaluk and Christison [2], horses do not extensively profit from shelters built to minimize chilling in cold weather or discomfort in hot weather. Behavioural and physiological (rectal and skin temperature, respiration rate, sweat score) studies performed on horses in a hot, sunny environment showed that horses prefer shade when it is available [17,18,19]. However, the outcomes did not lead to the conclusion that the provision of shade is an absolute minimum care requirement. 

The horse’s welfare and, consequently, the emotional arousal may be connected with thermally stressful conditions. The excitability state may be analysed with regard to the physiological changes in cardiac activity and psychological changes manifested by behaviour [20,21,22]. The cardiac activity variables have not been monitored in studies on using shade by horses as yet, whereas the use and effect of mist curtains have not been scientifically studied in horses at all. It was hypothesised that domestic horses released freely into a paddock do not show a distinct preference to cool in shady places or tap water under a mist curtain during high ambient temperature and intense solar radiation. The aim of the study was to determine whether horses under heat conditions during a 45 min turnout show behavioural or physiological symptoms of thermal discomfort and if their behaviour and cardiac parameters are related to freely chosen insolated, shaded, or water-sprayed areas in the paddock.

## 2. Materials and Methods

### 2.1. Horses

The study included six mares and six geldings. They were adult, warmblood horses usually used in leisure riding for one hour daily. None of the horses showed any health disturbances. The mares selected for the experiment were barren and not in oestrus. All of the horses lacked behavioural disturbances and excessive excitability, which was noted in a test of an unfamiliar person performed by a behaviourist before the study [23]. The horses were maintained in 3 × 3 m box-stalls. They were turned out into the paddock for two hours per day, alone or with a conspecific. The horses were habituated to washing. Their limbs below the carpal and hock joints were washed after the riding hour throughout the year, whereas the neck, back, and chest were additionally washed with tepid water on summer days. During the experimental day, the horses were not used for riding. 

### 2.2. Experimental Procedure

The 40 × 45 m experimental paddock was sand-covered and fenced with metal horizontal bars. It was located 3 m from the stable, away from roads or noise. Water and food were not available in it and no green growth around the paddock was present. Trees with highly elevated branches (not available to touch by the horses) shaded around 45% of the surface in the paddock. The horses were familiar with this paddock. An automatic mist curtain (UltraMist, Baszkówka, Poland) (250 cm height and 250 cm width) was installed one week before the experiment in a paddock corner which was insolated all the time. It was possible for the horses to go around the curtain. The temperature of the tap water in the curtain was approximately 16 °C. The horses were led under the curtain three times each day in the week before the test to make them accustomed to it. It was observed that afterwards, the horses did not avoid the curtain but were familiar with it.

A sport-tester (Polar ELECTRO OY-RS800CX sport140, Polar Electro Oy, Kempele, Finland) to measure the heart rate variability (HRV) was attached to each horse before its release into the paddock to familiarise the horse with the device for one week before the beginning of the experiment. The sport-tester was attached to an elastic belt placed around the chest of the horse and the tester was activated manually. Next, the horse was left alone in the box for approximately 5 min to get accustomed to the device. 

The experiment was carried out for three days at the end of July when the weather conditions were similar. Four horses per day were tested. Each studied horse was equipped with a halter on the head and an activated sport-tester attached to the trunk. The solitary horses were randomly, individually tested in the afternoon hours during intense solar radiation. On the day of the study, the first solitary horse was turned out to the paddock for 45 min at 12:00, the next one at 13:00, another at 14:00, and the last one at 15:00. The test did not last long to avoid important changes in the weather conditions. In the paddock, the horse could freely move and choose a preferred area: insolated (IS), shaded (SH), or under the mist curtain located all the time in the sun (MC).

The experimenter was not visible to the horse. The test was continuously recorded in videos using four cameras for a later detailed analysis of the horses changing areas in the paddock and their behaviour.

### 2.3. Weather Conditions

The experiment was performed on hot and sunny days. The atmospheric conditions were estimated using the psychrometric method. The average air temperature during the hours of the tests was 29–32 °C in the shade and the perceived temperature determined based on ambient temperature, wind speed, and humidity was 31–34 °C. The wind speed was up to 0.5 ± 0.1 m/s and the humidity was 42.0 ± 1.5%. 

### 2.4. Data

The data recorded throughout the experiment were analysed. A horse’s preferences for staying in one of the three areas, each offering different conditions (IS, SH, and MC) in the paddock were first determined based on the time the horse spent in the paddock. The percentage of time spent in each of the three areas was analysed in the video films and based on the analysis of the time axis in the figures automatically shown by the software. Since the time of staying in a given area by a horse was recorded in seconds and totalled, the other studied parameters could be considered for the time spent by the horses in each of the three areas separately. The changing border of IS and SH areas in the paddock was continuously taken into account according to the video recordings. It was assumed that the horse was present in a given area when at least its head, neck, and withers were in it. 

The emotional arousal of horses in response to staying under different paddock conditions was considered in terms of differences in cardiac activity: heart rate (HR; beats per minute, bpm) and heart rate variability (HRV). The HR and HRV data recorded with sport-testers were downloaded to a computer with a peripheral IrDA USB 2.0 adapter. The analysis was conducted with PolarProTrainer 5.0 (Polar Electro Oy, Kempele, Finland) and Kubios HRV 2.0 software (University of Kuopio, Kuopio, Finland) [21,24]. Since the HRV analysis was based on the heart beat-to-beat intervals (RR), the RR recordings were first adjusted to eliminate artefacts and the primary registered cardiac rhythm was controlled to filter out stimulation originating from conduction centres other than the sinoatrial node. The low correction factor of the custom filter was used. The individual RR values identified by the programme as artefacts were automatically substituted by interpolated intervals calculated from the difference between the previous and the next value accepted by the programme RR values. Using this filter was sufficient to eliminate artificial, single peaks in RR recordings. The verified RR values (normal inter-beat intervals; NN; presented in milliseconds; ms) were used for further analyses. HR and the following HRV variables were analysed:

(1) NN;
(2) the root mean square of the successive differences in beat-to-beat intervals (RMSSD; ms);
(3) the low-frequency component of the power spectrum assigned to the tone of the sympathetic nervous system (LF; Hz, ms^2^), which ranges from 0.04 Hz to 0.15 Hz;
(4) the high-frequency component of the power spectrum assigned to the tone of the parasympathetic nervous system (HF; Hz, ms^2^), which ranges from 0.15 Hz to 0.40 Hz and represents the activity of the parasympathetic nervous system;
(5) the ratio of spectrum density power from low to high frequencies (LF/HF).

HR, LF, and the LF/HF increase as a result of sympathetic nervous system activity, whereas NN, RMSSD, and HF reflect the predomination of parasympathetic activity. 

The data recorded during the 1 min periods of the test were analysed. The time point of each period was determined based on an analysis of the time axis in figures automatically shown by the software. The variables were considered during all 1 min periods spent by horses entirely in a given area. The periods within which a horse changed areas were not considered.

The behaviour of the horse during the test was classified into standing or locomotor activity: single steps, walk, trot, and canter [25]. More than three steps without interruption were recorded as the gait. One to three steps were assumed to be single steps. These behaviours were considered exclusively in terms of time, i.e., in the percentage of time spent in the experimentally different paddock areas (IS, SH, or MC). Moreover, two non-locomotor behaviours were taken into account: rate of vocalisations [26] and yawning [27] per min of staying in these areas. The types of calls were not distinguished since their prevalence was low. Other behaviours were too rare to be statistically considered.

In addition, the horse rectal temperature before and immediately after the test was measured with a veterinary thermometer Veterinär—Thermometer SC 12 (measurement time: 30 s). The effect of time spent by horses in experimentally different conditions on rectal temperatures was analysed.

### 2.5. Statistical Analysis

The statistical evaluation of the data was carried out with Statistica 13.1 software and a Shapiro-Wilk test to check the normality of the data. According to this outcome, a repeated-measures ANOVA test with a post-hoc HSD Tukey test for multiple comparisons could then be performed to assess the changes in HR and HRV variables over different experimental conditions (IS, SH, MC). For the time of staying and behavioural parameters in horses present in various experimental conditions, the normality of data distribution was not confirmed, hence the influence of the treatment effect was estimated using a nonparametric Friedman test. A non-parametric Wilcoxon test was used to compare horse rectal temperature before and after the test. The dependence between the horse body temperature and time spent in different paddock areas was analysed with a Spearman rank correlation. The differences between means and correlations were considered significant at *p* < 0.05. 

## 3. Results

The Friedman test showed that the time spent by the horses in IS, SH, and MC did not statistically differ (*p* = 0.1161). The individual variation among horses shown by the standard deviation (SD) and coefficient of variation (V) was high (Table 1). The IS data were less dispersed than SH, whereas the highest variation concerned MC.

The Wilcoxon test showed that the mean rectal temperature in horses before (37.7 ± 0.23 °C) and after the test (37.6 ± 0.27 °C) did not significantly change (*p* = 0.9375). The temperature after the test was not significantly correlated with the time spent by a horse in different paddock areas (*p* > 0.05; Table 2).

**Figure 1:**
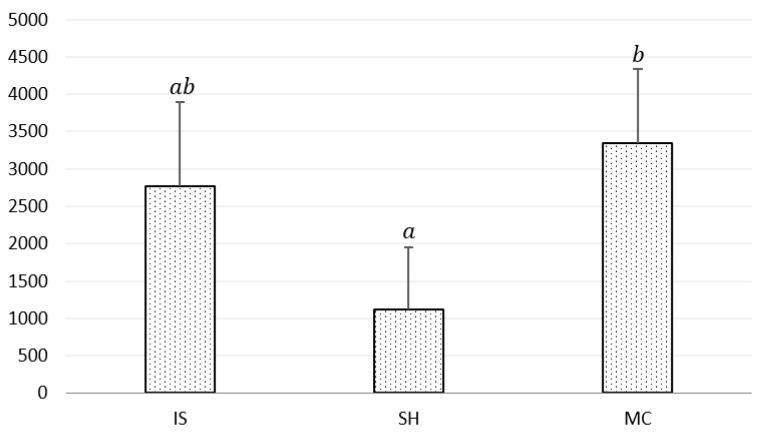
Mean HF with regard to paddock conditions: IS—insolated, SH—shaded, MC—under mist curtain. Vertical lines show the standard deviation. Means marked with different letters significantly differ at *p* < 0.05; the same letters indicate that significant differences do not appear.

An ANOVA test showed that, for cardiac variables, only HF was significantly different under experimentally different conditions (*p* < 0.05; Table 3). Tukey’s post-hoc HSD test revealed that HF obtained in MC was significantly higher than in SH (*p* = 0.03730), which is illustrated in Figure 1.

Table 4 presents the results of a Friedman test of behavioural parameters. Mean percentages of standing and particular gaits within the time spent under different paddock conditions did not significantly differ except for the walk (*p* < 0.05). The mean rate of yawning per min spent in these areas significantly differed (*p* < 0.05), whereas the rate of vocalisations was not statistically different.

**Figure 2:**
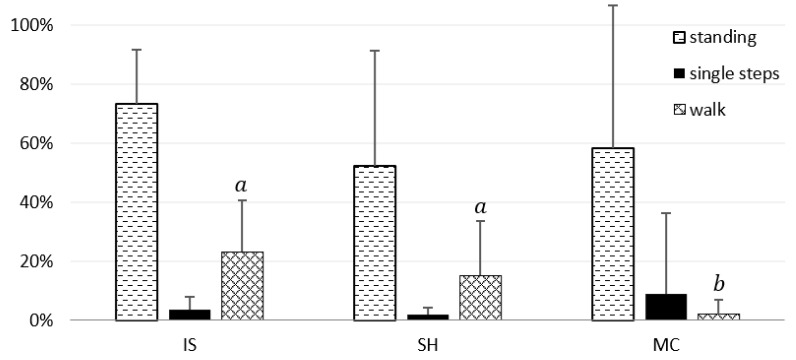
Percentages of time of standing, single steps, and walk with regard to the paddock conditions: IS—insolated, SH—shaded, MC—under mist curtain. Vertical lines show the standard deviation. Means marked with different letters within a kind of locomotor behaviour significantly differ at *p* < 0.05; the same letters or lack of letters indicate that significant differences do not appear.

Figure 2 shows that the percentage of time of standing amounted to 73.2 ± 18.5%, 52.1 ± 39.0%, and 58.4 ± 48.3% in IS, SH, and MC, respectively. The locomotor behaviours were rare and consisted almost exclusively of walking and single steps. The percentage of time of walking was higher in IS (23.2 ± 17.3%) and SH (15.1 ± 18.4%) than in MC (1.9 ± 4.72%; *p* < 0.05). Single steps occurred during 3.6 ± 4.4% of the time spent in IS, 1.8 ± 2.5% in SH, and 8.8 ± 27.4% in MC. The trot and canter were very rare in IS (0.02 ± 0,07%; 0.01 ± 0.02%, respectively) and SH (0.06 ± 0.15%; 0.18 ± 0.44%, respectively), although they did not occur in MC.

**Figure 3:**
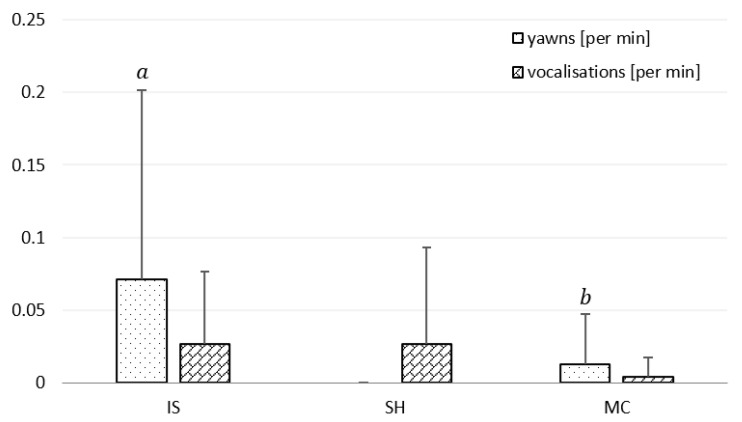
Mean rate of yawns and vocalisations per min with regard to the paddock conditions: IS—insolated, SH—shaded, MC—under mist curtain. Vertical lines show the standard deviation. Means marked with different letters within yawns significantly differ at *p* < 0.05; no letters indicate that significant differences do not appear.

Yawns were the most frequent in IS (0.071 ± 0.13 per min), less frequent in MC (0.013 ± 0.03 per min) and did not occur in SH (*p* < 0.05; Figure 3). The rate of vocalisations was similar between particular areas: 0.004 ± 0.04 per min in MC to 0.026 ± 0.05 per min in IS and 0.026 ± 0.07 per min in SH. 

## 4. Discussion

Horses are often released to paddocks for a short time after work. The answer to whether they are tolerant to high ambient temperature in the afternoon (and solar radiation) is important for providing them with sufficient welfare conditions. The limitation of the study is that solar radiation dropped with consecutive experiment hours. It does seem, however, that the changing angle of solar radiation within those hours (4.5°/hour) could not be a great difference perceived by the horses. The analysis reveals that during a short, 45 min turnout under heat conditions, the horses did not show distinct preferences for IS, SH, or MC area. Moreover, the rectal temperature remained unchanged, regardless of the time spent under different conditions. According to Holcomb et al. [17,18], horses spend more time in the shade than just by chance alone. The authors found that when horses were studied for five days, their preference for shade was greatest before and during peak solar radiation and then again, despite expectation, several hours later following peak black globe temperature [17]. Another study conducted for 40 days showed that horses used shade more than just by chance on the sunniest days at all hours of the day with the greatest use in the late morning [18]. The current results may be different because the horses were turned out for a short time. It appears that within 45 min, the studied conditions may be somewhat unchanged to note any effect upon the horses’ perception or physiology.

Tap water mist produced by a curtain may cause immediate short-term constriction of the vessels accompanied by an increase in the core temperature. Vasodilation and heat dissipation begins after this initial phase [28]. The sudden body reactions may elicit unpleasant or sudden feelings which could result in very weak locomotor activity in MC in the horses of the present study. Only single steps tended to occur more often under those circumstances. The sudden and unexpected feelings could have been one of the reasons for great individual differences in the horses using the mist curtain. In the study, the lack of a significant difference between the time spent in IS, SH, and MC probably resulted from the dispersion of the data: three horses stayed in MC for over 2000 s, whereas four other horses did not enter the mist curtain at all. However, similar time spent by the horses in SH and MC is noteworthy and may indicate the benefits of locating mist curtains in paddocks.

The locomotor activity mostly included walking first of all in IS and SH and, as has been mentioned, seldom in MC. The locomotor activity of free-ranging horses mainly results from the need for searching for food. Canter is associated with fleeing in situations of predator stalking or other threats. Although horses living in nature move and graze most of the day, apart from grazing, their daily locomotion lasts for a short time [29]. Walking comprises 3–10% of their time and trotting or cantering less than 1% [15]. Stabled horses released into a paddock do not experience such needs, hence they spend still less time in locomotion [14,30]. The current results are consistent with those findings. The low frequency of locomotor behaviours could have also resulted from the high ambient temperature. It seems that the locomotor activity in the horses turned out to the paddock was too low to elicit any cardiac changes. 

The rates of vocalisation and yawning were low. Specific types of calls may be associated with positive or negative situations. Vocal expression of emotions is thought to be conserved throughout evolution [31]. According to Stomp et al. [32], signals produced by the nostrils during expiration (snorts, snores, and blows) occur without a relationship with air conditions. The lack of differentiation in the rate of vocalisations in different paddock areas in the current study is similar to that finding. 

Yawning occurred mainly in IS and was rarer in MC, whereas it did not appear in SH. Studies on the yawning frequency proved several factors eliciting this behaviour connected with, among others, excitatory or stressful social situations [27], frustration and stereotypic behaviour [33], alertness or drowsiness state [34], as well as thermoregulation and brain cooling [35,36]. The higher rate of yawning in IS in the current study could have been mainly caused by the drowsiness state of the horses, thermoregulation, and brain cooling, since the horses were turned out in solitary and no stressful situations occurred. 

The analysis of the HR and HRV parameters in the studied horses showed that there were generally no differences within the variables related to the freely chosen place of staying in the paddock. It means that the horses showed the same level of emotional arousal staying in IS, SH, or MC. The only exception was HF, which was significantly higher in both IS and MC than in SH. Since an increase in HF indicates a shift towards the dominant activity of the parasympathetic nervous system, the IS and MC values of this parameter are characteristic of the relaxation state of the horses [20]. Elevated values of HF were reported in horses subjected to listening to relaxing music and/or to relaxing massage [37,38]. The current results may indicate that horses were slightly more relaxed staying in IS or MC than in SH. This statement seems to be consistent with the more frequent yawning in IS which, as mentioned, was not a result of stressful situations but rather calming factors. The reduced HF values stated in SH could have been partly caused by flying insects biting and annoying the horses. Holcomb et al. [17] found that although there was no difference in the insect count in shaded and unshaded areas, horses showed a trend toward more insect avoidance behaviour (head movements and hoof stomping) in the shaded area. However, an earlier study by Holcomb et al. [39] did not reveal such a trend. 

To sum up, the analysis of changes in HR and HRV parameters (especially HF values) indicated that horses did not show any signs of overheating but were rather tolerant of the experimental conditions. The results indicate that the IS, SH, and MC conditions in the paddock do not clearly affect the preferences regarding the time spent, behaviour, or the physiology of the horses (the latter in terms of cardiac and nervous system activity changes). It appears that the horses felt well in IS under heat conditions since they stayed there for a time similar to areas offering other conditions, and they walked and yawned more frequently. It can be suggested that the horses which displayed a higher ability of thermoregulation in IS remained in IS, whereas horses that felt discomfort in IS more often used SH. This could be a reason for the higher activity of the sympathetic nervous system (shown by HF) in horses staying in SH. The similar parameters studied in different paddock conditions probably result from the possibility of free choice of the area by the horses—when the horses feel uncomfortable in a certain area, they change it into another one. This can help to maintain the variables at a similar level. In the opposite case, when horses are confined in a sunny environment, both their rectal and skin temperatures increase compared to horses confined in the shade, which was found by Holcomb et al. [39].

The current study shows that the horses’ preferences regarding the conditions in the paddock during heat are not evident. This may result from high resistance to the heat of non-exercised but freely moving horses, particularly during a short turnout. The behaviour of horses freely moving outdoors and seeking IS, SH, or MC is important when planning paddocks and pastures in a facility. Future studies should reveal whether the behaviours and physiological measures of the horses recorded in this study are similar at different times of the day and if horses prefer to use the mist curtain instead of the shade during a longer turnout. 

## 5. Conclusions

Horses turned out for 45 min in a hot environment do not show distinct preferences for staying in IS, SH, or MC, although individual differences between horses are great. Horses staying under IS and MC conditions show a higher level of relaxation expressed by higher HF compared to those of horses staying in SH. Horses do not exhibit symptoms of thermal discomfort during staying in IS for such a short turnout. The free choice of one of the areas, each offering different environmental conditions, may be a crucial factor in maintaining body temperature as well as emotional arousal (manifested by cardiac variables and expressed by locomotor activity) with the rates of various behaviours at similar levels. Thus, the provision of a shade and mist curtain in paddocks seems to be reasonable. The response of horses to spending longer periods in IS, SH, and MC requires further investigation.

## Figures and Tables

**Table 1:** Mean time (s) spent by the horses in different paddock areas during 2700 s of the test. Means do not significantly differ (*p* > 0.05).

Paddock Conditions	Mean	SD	V [%]	% of Test Time
IS	1259.8	880.9	70	46.7
SH	719.4	927.2	129	26.7
MC	721.5	968.0	134	26.7

IS—insolated, SH—shaded, MC—under mist curtain; SD—standard deviation; V—coefficient of variation.

**Table 2:** Spearman rank correlation between rectal temperature after the test (°C) and time spent by the horses in different paddock areas during 2700 s of the test. Correlations are not significant (*p* > 0.05).

Paddock Conditions	IS	SH	MC
Correlation	0.2556	−0.2752	0.3335
*p*-value	0.4091	0.1232	0.5061

IS—insolated, SH—shaded, MC—under mist curtain.

**Table 3:** The influence of experimental conditions on HR and HRV parameters: ANOVA results.

Parameter	*p*-Value
HR	0.0893
NN	0.0512
RMSSD	0.1321
LF	0.0587
HF	0.0382 *
LF/HF	0.7315

* *p* < 0.05.

**Table 4:** The influence of experimental conditions on behavioural parameters: Friedman test probability value.

Behavioural Parameters	*p*-Value
Standing [%]	0.5837
Single steps [%]	0.1253
Walk [%]	0.0038 *
Trot [%]	0.2725
Canter [%]	0.3679
Vocalisations [per min]	0.4308
Yawns [per min]	0.0096 *

* *p* < 0.05.

## Data Availability

The data presented in this study are available on request from the corresponding author.
